# Cervicovaginal Microbiome after Cervical Intraepithelial Neoplasia Treatment. A Protocol for Systematic Review and Meta-Analysis

**DOI:** 10.3390/ijerph18179050

**Published:** 2021-08-27

**Authors:** Marta Janicka-Kośnik, Beata Sarecka-Hujar, Grzegorz Jakiel, Aneta Słabuszewska-Jóźwiak

**Affiliations:** 1 Saint Sophia Hospital, Żelazna 90 Street, 01-004 Warsaw, Poland; 2 Department of Basic Biomedical Science, Faculty of Pharmaceutical Sciences in Sosnowiec, Medical University of Silesia in Katowice, Kasztanowa 3 Street, 41-200 Sosnowiec, Poland; beatasarecka@poczta.onet.pl; 3 First Department of Obstetrics and Gynecology, Centre of Postgraduate Medical Education, Żelazna 90 Street, 01-004 Warsaw, Poland; grzegorz.jakiel1@o2.pl (G.J.); anetaslabuszewska@gmail.com (A.S.-J.)

**Keywords:** microbiome, cervicovaginal microbiome, intraepithelial neoplasia, cervical cancer, intraepithelial neoplasia treatment, LEEP

## Abstract

(1) Background: The microbiome consists of microorganisms from various kingdoms with numerous physical and chemical properties Lactobacillus species constitute the highest percentage of healthy cervical and vaginal microbiota. Dysbiosis may cause adverse outcomes, e.g., bacterial vaginosis, pelvic inflammatory disease and pregnancy complications. The cervicovaginal microbiome might contribute to the development of a persistent HPV infection—the main risk factor of cervical cancer—and influence progression to malignancy The aim is to perform a systematic review of current literature and a meta-analysis regarding microbiome changes after cervical intraepithelial neoplasia treatment. (2) Methods: We will search PubMed, Scopus, Google Scholar and Embase Database and trace citations in the reference sections. Randomized and non-randomized controlled studies, case–control and cohort studies published between January 2000 and May 2021 will be included in the study protocol. The following keywords will be used: ‘microbiome’, ‘vaginal microbiome’, ‘cervical microbiome’, ‘cervical neoplasia treatment’, ’conization’, ‘electroconization’, and ‘electrosurgical treatment’. Statistical analyses will be performed using RevMan 5.4. (3) Results: The results will be published as a peer-reviewed article. (4) Conclusions: The study will show which forms of intraepithelial neoplasia treatment change the cervicovaginal microbiome. Finding the best form of treatment by studying the cervicovaginal microbiome after various forms of treatment is essential. Patients would benefit not only from the treatment of the initial disease but also the management of dysbiosis, which might underlie other pathologies.

## 1. Introduction

The precise definition of the human microbiome was presented by Berg et al. in 2020. It was described as a group of microorganisms originating from various kingdoms, occurring in specific areas, with an array of physical and chemical properties [1]. The female lower reproductive tract is an area where microorganisms are present at high concentrations, with Lactobacillus species constituting the highest percentage of healthy cervical and vaginal microbiota [2]. Dysbiosis, which is an imbalance between the species of microorganisms, may contribute to the development of numerous adverse outcomes. The most common ones associated with the genitourinary tract are bacterial vaginosis and pelvic inflammatory disease, but also negative pregnancy outcomes such as miscarriage and preterm birth [3]. The diagnosis and impact of the cervicovaginal microbiome on gynecologic neoplastic changes are a new field under investigation. The risk factors contributing to dysbiosis, such as nicotine use, contraceptive use and parity, were correlated with malignancy in the genitourinary tract [4,5]. Cervical cancer is now ranked the seventh cancer in terms of causing death in women in Poland. Human papilloma virus (HPV) is the most common factor responsible for the development of cervical intraepithelial neoplasia lesions. Some studies highlighted the importance of the microbiome in the development of a persistent HPV infection and its influence on progression to malignancy [2,4,6]. Some authors were hesitant whether an HPV infection was the cause of dysbiosis or dysbiosis was a risk factor of developing a persistent HPV infection [4]. It is possible that the treatment of cervical intraepithelial neoplasia might lead to microbiome transformation. In this protocol for systematic review and meta-analysis, we would like to introduce a study of the cervicovaginal microbiome after cervical intraepithelial neoplasia treatment. The analysis of articles retrieved from various databases will provide us with information, based on which statistical analysis will be conducted to confirm how the form of treatment of intraepithelial neoplasia changes the cervicovaginal microbiome.

## 2. Materials and Methods

### 2.1. Protocol Register

This protocol of systematic review and meta-analysis has been drafted under the Preferred Reporting Guidelines for Systematic review and Meta-analysis Protocol (PRISMA) [7] and registered on the International Prospective Register of Ongoing Systematic Reviews (PROSPERO) [8], registration number CRD42021255805.

### 2.2. Ethics

The approval of an ethics committee is not required for this protocol. Data are not individualized.

### 2.3. Inclusion Criteria

1. Randomized and non-randomized controlled studies, case–control and cohort studies.

2. Articles posted on PubMed, Google Scholar, Scopus and Embase Database;

3. Articles in English;

4. Articles published between January 2000 and May 2021—this period is characterized by the recent introduction of current diagnostic technique;

5. Patients with cervical intraepithelial neoplasia treated with LEEP (loop electrosurgical excision procedure), cold knife conization, classic surgery or laser conization;

6. Patients with dysbiosis and a persistent HPV infection treated with LEEP (loop electrosurgical excision procedure), cold knife conization, classic surgery or laser conization.

### 2.4. Exclusion Criteria

1. Articles in any language other than English, published earlier than January 2000, posted on other science websites;

2. Papers without complete data, not completed even after the authors were contacted;

3. Animal studies, letters and comments, review articles and editorials;

4. Studies related to the microbiome and dysbiosis peri- and postmenopausal women;

5. Studies concerning the microbiome and dysbiosis after radio- and chemotherapy and fertility treatment;

6. Studies that include women with a recurrent HPV infection and or squamous epithelial lesions;

7. Studies related to the microbiome and dysbiosis HIV-positive women.

### 2.5. Information Sources and Search Strategy

We will search PubMed, Scopus, Google Scholar and Embase Database. We will also trace citations in the reference sections. Randomized and non-randomized controlled studies as well as case–control studies published between January 2000 and May 2021 will be included in the study protocol. The following key words will be used in the searching process: ‘microbiome’, ‘vaginal microbiome’, ‘cervical microbiome’, ‘cervical intraepithelial neoplasia treatment’, ’conization’, ‘electroconization’, and ‘electrosurgical treatment’. Study titles and abstracts will be screened according to the inclusion/exclusion criteria. We will cross-check the cited references as well. The selected relevant studies will be classified according to the population, intervention, comparison and outcome (PICO) framework in order to identify the relevant research questions meeting the following selection criteria: 

Population: women with cervical intraepithelial neoplasia;

Intervention: conization;

Comparison: women with a PAP smear not raising any suspicions with a negative HPV test;

Outcomes: microbiome changes.

### 2.6. Data Filtering and Extraction

Two authors of this protocol will search all included databases separately. The authors will exclude all studies that do not meet the inclusion criteria. Subsequently, abstracts and full articles will be excluded if they do not meet the criteria. All data will be extracted. Any disagreement will be solved by consulting the third researcher. The process of literature searching will be presented as a flow diagram according to the PRISMA Statement [9].

### 2.7. Missing Data

If any important data are missing in the searched articles, the authors of this protocol will contact the author of the chosen article directly via correspondence. 

### 2.8. Literature Quality Assessment

Randomized and non-randomized controlled studies involved in the meta-analysis will be assessed according to the Cochrane Handbook for Systematic Reviews of Interventions available from www.training.cochrane.org/handbook, accessed on 24 June 2021. The quality of cohort studies will be assessed with the Newcastle Ottawa Scale [10] for cohort studies. All disagreements will be resolved by discussion.

### 2.9. Assessment of Reporting Biases

The authors will independently assess the risk of bias for each study using the criteria outlined in the PEDro scale [11] for randomized studies and the Newcastle Ottawa Scale (NOS) [10] for cohort studies. The PEDro scale evaluates 11 items: inclusion criteria and source, random allocation, concealed allocation, similarity at baseline, subject blinding, therapist blinding, assessor blinding, completeness of follow-up, intention-to-treat analysis, between-group statistical comparisons, and point measures and variability. Each item will be rated as ‘yes’ or ‘no’, and the total PEDro score will be the number of criteria met (excluding the inclusion criteria and the source item). Articles with the scores ≥ 7 points on the NOS scale (the scale of 0–9) will be considered high-quality ones. Any disagreement between the two researchers will be resolved by consensus or by consultation with the third researcher.

### 2.10. Statistical Analysis

Statistical analyses will be performed using the Review Manager software (RevMan version 5.4 Cochrane, London, UK) and MedCalc software (version 19.5.3.; MedCalc Software Ltd., Ostend, Belgium). To determine the association between the form of treatment for intraepithelial neoplasia and the changes in the cervicovaginal microbiome, the pooled odds ratios (OR) with the 95% confidence intervals (CI) will be calculated.

#### 2.10.1. The Analysis of Heterogeneity

The heterogeneity between studies will be assessed with the use of the I^2^ test. I^2^ expresses the proportion of dispersion due to heterogeneity I^2^ at 25%, 50% and 75% will suggest low, intermediate, and high inconsistency, respectively. We will assume that I^2^ at the level of 50% will allow us to select between using the random-effects model (REM) or the fixed-effects model (FEM) to calculate the pooled OR along with a 95% CI. 

#### 2.10.2. Publication Bias

The publication bias will be evaluated both visually with the inspection of funnel plots and by performing the Egger’s test as well as the Begg’s test. A minimum of 10 studies should be included to conduct funnel plot asymmetry tests so as to maintain sufficient power for distinguishing the chance from real asymmetry.

#### 2.10.3. Subgroup Analysis 

If we identify substantial heterogeneity, we will investigate it using subgroup analyses. 

#### 2.10.4. Sensitivity Analysis

Sensitivity analysis will be performed to assess the stability of the results. During this analysis, a single study will be omitted at a time to reflect the influence of the individual data set on the pooled OR. Leave-one-out analysis involves performing a meta-analysis on each subset of the studies obtained by leaving out exactly one study. It shows how each individual study affects the overall estimate of the remaining studies. Comparisons of the results obtained in the systematic review with the raw data from the individual publications will be shown as forest plots after calculating pooled OR values using random or fixed effects model and in turn will be discussed in the Discussion section.

## 3. Results

The results of the analysis will be investigated according to race, parity and age. The results will be published as a peer-reviewed article. 

## 4. Discussion

An increasing number of studies tackle the problem of cervicovaginal microbiome dysbiosis. It is a field that still needs to be investigated. A lot of evidence is available to support the fact that the microbiome may lead to the persistence of an HPV infection and result in cervical neoplasia [4]. Coping with an HPV infection and the subsequent carcinogenesis may be associated with a balanced microbiome and its fight mechanisms [6,12]. Finding the best form of treatment by studying the cervicovaginal microbiome after various forms of intraepithelial neoplasia treatment is a new approach. Patients might benefit from it not only because of the treatment of the initial disease but also due to the management of dysbiosis, which may underlie numerous other pathologies.

### Study Limitations

It is possible that the final conclusions will be incomplete due to the limited number of studies as well as a limited number of participants in the studies, Moreover, the interpretation of statistical analysis might be difficult due to various approaches to this subject by the authors of available articles. The analysis of the patients’ microbiome might be performed with different methods, which might provide a spectrum of results. It is possible that further studies on larger groups will be essential to form direct conclusions.

## 5. Conclusions

We hope that this study will provide information on how the form of the treatment of intraepithelial neoplasia changes the cervicovaginal microbiome. This knowledge is a continuation of the attempt at understanding the complex problem of cervical intraepithelial neoplastic changes and might help to indicate the best form of treatment according to evidence-based medicine. 

## Data Availability

Data available in a publicly accessible repository that does not issue DOIs. Publicly available datasets were analyzed in this study. This data can be found here from 2 May 2021: [https://drive.google.com/drive/folders/19aPIJnrXTsvwKPUVCn0NtauduGuFgeC4?usp=sharing].
